# Near-field collimation of light carrying orbital angular momentum with bull’s-eye-assisted plasmonic coaxial waveguides

**DOI:** 10.1038/srep12108

**Published:** 2015-07-10

**Authors:** Mingbo Pu, Xiaoliang Ma, Zeyu Zhao, Xiong Li, Yanqin Wang, Hui Gao, Chenggang Hu, Ping Gao, Changtao Wang, Xiangang Luo

**Affiliations:** 1 State Key Laboratory of Optical Technologies on Nano-Fabrication and Micro-Engineering, Institute of Optics and Electronics, Chinese Academy of Science, Chengdu 610209, China

## Abstract

The orbital angular momentum (OAM) of light, as an emerging hotspot in optics and photonics, introduces many degrees of freedom for applications ranging from optical communication and quantum processing to micromanipulation. To achieve a high degree of integration, optical circuits for OAM light are essential, which are, however, challenging in the optical regime owing to the lack of well-developed theory. Here we provide a scheme to guide and collimate the OAM beam at the micro- and nano-levels. The coaxial plasmonic slit was exploited as a naturally occurring waveguide for light carrying OAM. Concentric grooves etched on the output surface of the coaxial waveguide were utilized as a plasmonic metasurface to couple the OAM beam to free space with greatly increased beam directivity. Experimental results at λ = 532 nm validated the novel transportation and collimating effect of the OAM beam. Furthermore, dynamic tuning of the topological charges was demonstrated by using a liquid crystal spatial light modulator (SLM).

In the modern theory of quantum physics, light possesses a range of properties that are historically thought to belong to particles and waves, respectively. The understanding of the relation between the particle-like and wave-like properties is of both fundamental and practical interest^1^,^2^. As one of the particle-like properties of light, the optical momentum manifests itself in the mechanical interaction with matter, i.e., the momentum transfer between light and the objects^3^,^4^.

Among the various kinds of momenta, the orbital angular momentum (OAM) of light has attracted special attention immediately after it was discovered by Allen *et al.* in 1992^5^. The OAM beam is typically characterized by a doughnut-shaped intensity profile associated with an azimuthal phase term exp(i*ℓφ*), i.e., a momentum of *lћ* per photon at a quantum level. Nowadays, the unique properties of OAM have been intensively studied and successfully applied in many realms such as optical tweezers^6^,^7^, microscopies^8^, and astronomy^9^.

In 2004, it was realized that OAM can provide an additional degree of freedom for data multiplexing in optical communications systems^10^. The orthogonality of the intrinsically unbounded OAM modes makes it possible to transfer information via each OAM channel. Unfortunately, the diffraction of OAM in free space will eventually lead to the rapid fall-off of the energy on the beam axis where the phase singularity exists. Therefore, it is argued that the OAM is an efficient communication protocol only when the propagation length is less than the Rayleigh length^11^. In this case, the OAM-based communication frame could be considered as a specific solution of the general multiple input multiple output (MIMO) techniques, where orthogonal multi-modes are exploited to enhance the capacity.

In the last several years, the interaction of light with structured material was extensively studied to generate the required OAM states. The diverse structures include liquid crystal spatial light modulator (SLM)^8^, space-variant antenna arrays^12^, ring resonators^13^, nano-wires^14^,^15^ and nano-holes^16^,^17^,^18^,^19^. Among these devices, plasmonic structures are of particular importance because the development requirement of integrated opto-electro system is becoming more and more urgent^20^,^21^,^22^,^23^,^24^. Owing to the extremely small effective wavelength^25^, the OAM modes in the nano-holes and similar plasmonic structures offer great opportunity to reduce the dimension of optical waveguides^26^,^27^. Nevertheless, the difficulty of efficient coupling between external light and the plasmonic waveguides is increasing along with the decrease of the waveguide size. As a result of the intrinsic diffraction, the focus spot of free-space OAM light may be much larger than the waveguide mode area, resulting in dramatically reduced coupling efficiency. Similarly, the divergence angle of the OAM beam radiated from the waveguide is inversely proportional to the size of the dielectric core. The mismatch between the waveguide and the light source poses a great challenge for practical on-chip applications.

Here we report the experimental observation of OAM coupling and transportation in plasmonic coaxial waveguides. Owing to the subwavelength core width, extremely large topological charges were demonstrated to be able to transfer in the waveguides. With the aid of circular grooves at the output surface of the waveguide, the guided plasmonic OAM beam was first converted to spiral surface plasmon polariton (SSPP) and then scattered into the far-field. Scanning near-field optical microscopy (SNOM) was utilized to detect the optical fields directly in the near field. By employing liquid crystal SLM as an active element, dynamic change of the topological charge was also enabled.

## Results

### Principle and numerical results

It is well known that coaxial waveguides in the microwave regime are able to transfer radially polarized modes carrying OAM, whereas their optical counterparts have long been prohibited by the large ohmic loss in the metallic structures. In recent years, it was demonstrated that the plasmonic coaxial waveguides could be used in some special cases by sacrificing the propagation length^28^,^29^,^30^,^31^,^32^. Since light is not confined by total internal reflection, sharp bending of wave is possible. Nevertheless, it seems surprising that little work has been reported regarding the OAM transfer in plasmonic coaxial waveguide^26^. In the following, we show that the coaxial OAM waveguide is able to support the steady propagation of OAM light. With the help of periodic shallow grooves (also known as a type of metasurface^25^), we demonstrated that the OAM waveguide modes could be efficiently coupled to free-space, accompanied with an enhanced directivity. This new technique can provide much larger degree of freedom compared to fiber-based OAM waveguide^33^ because more OAM modes could be utilized in a single waveguide.

**Figure 1:**
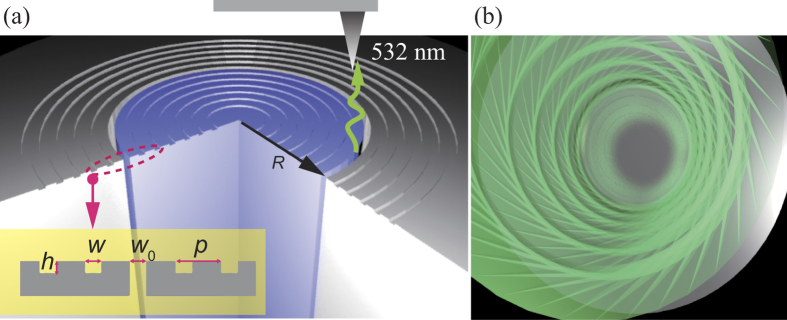
Coaxial waveguide for guiding and collimating OAM beam. (**a**) Sketch of the waveguide, with grooves etched on the output surface. (**b**) Schematic diagram of the collimating effect of the OAM beam in free-space.

The schematic of the plasmonic OAM waveguide is illustrated in Fig. 1(a), where periodic shallow grooves are etched on the metallic surface with a width of *w*, depth of *h*, and period of *p*. The dielectric core is placed at the center of these grooves, with a width of *w*_0_ and radius of *R*. The whole structure is referred to as a reformed bull’s eye, following the definition in previous proposals^20^,^24^.

In essence, this plasmonic coaxial waveguide could be treated as a rounded metal-insulator-metal (MIM) waveguide^20^, where the polarization state changes from transverse magnetic (TM) to radial polarization, i.e., all the electric field vectors are polarized along the radial direction^30^. After the radially-polarized OAM beam is emitted out from the central slit, SSPP would be excited at the metal-dielectric surfaces and then coherently scattered by these grooves. As depicted in Fig. 1(b), the superposition of the directly emitted OAM and scattered SSPP could become highly collimated. In principle, the *z*-component of the electric field in the SSPP could be written in a simple form as follows:
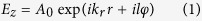
where *A*_0_ is the amplitude, *l* is the topological charge of the OAM beam, *k*_*r*_ is the wavevector along the radial direction, which can be calculated by the relation:
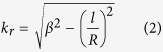
Here *β* is the wavevector of the SSPP at the metal-dielectric surface^25^:
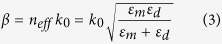
where *ε*_*m*_ and *ε*_*d*_ are the dielectric constants of the metal and dielectric, *n*_eff_ is the effective refractive index, *k*_0_ is the wavevector in free space. In this paper, the metal is chosen as silver (Ag) and dielectric is air. At a wavelength of λ = 532 nm^34^, the calculated effective refractive index is 1.0524 and the effective wavelength is 505.5 nm.

The coherent scattering of SSPP at the periodically placed grooves can increase the effective radiation width. As a result, the angular width of the radiation pattern would decrease correspondingly. Interestingly, we find that the reduction of the divergence angle in the bull’s eye is not only resulted from the increased effective radiation aperture, but also from the contribution of super-oscillatory field, which is a universal phenomenon in electromagnetics as first recognized by Toraldo in 1952^35^.

**Figure 2:**
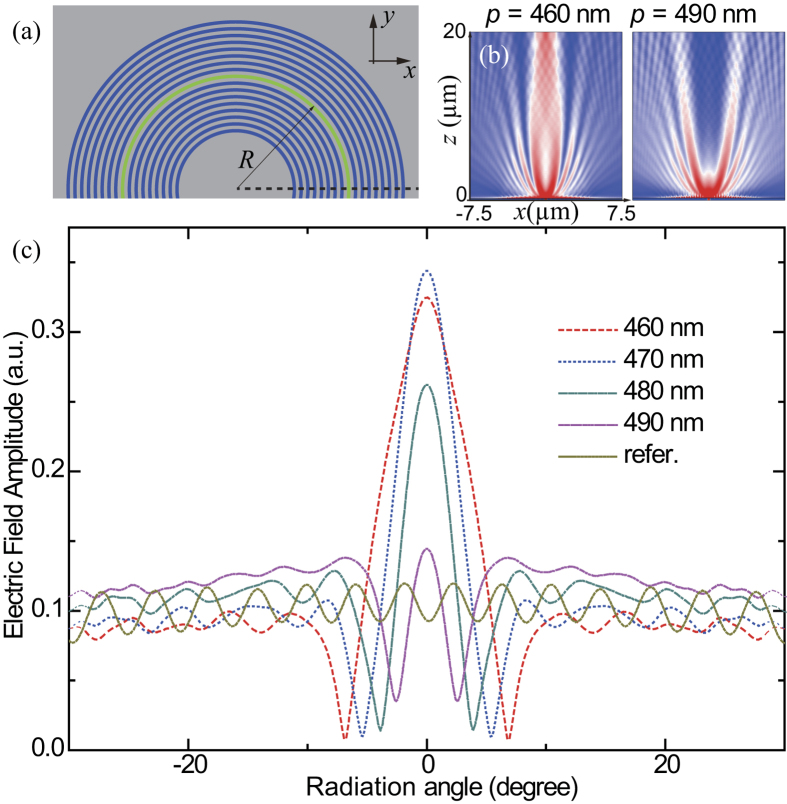
Super oscillation and the beaming effect. (**a**) Sketch of the circular slit surrounded by both inner and outer grooves. (**b**) Magnetic field intensity distributions at the *xz* plane for *p* = 460 nm and 490 nm. (**c**) Far-field electric field amplitudes calculated by Fourier transformation. The radiation angle is defined with respect to the *z*-axis.

As shown in Fig. 2(a), 16 concentric grooves were etched on the two sides of the dielectric core symmetrically. To investigate the impact of geometric parameters on the beaming performance, we focused on the two-dimensional (2D) counterpart of this structure, as indicated by the dashed line along the radial direction. The groove width *w* and height *h* were set as 230 nm and 40 nm. The slit radius *R* and width *w*_0_ were chosen as 7.5 μm and 230 nm, respectively. Under TM polarized illumination, the magnetic field distributions in the *xz* plane (Fig. 2(b)) validated the beaming effect, coinciding well with the initial results given by Ebbesen *et al.*^20^. Figure 2(c) represents the radiation pattern for varying periods between 460 nm and 490 nm (the step is chosen as 10 nm), where the angular full width at half maximum (FWHM) is 8.6°, 5.9°, 4.5°, and 3.6°, respectively.

The most obvious sign of the super-oscillation effect is that the reduction of the angular FWHM is accompanied with the increase of side lobe. As a result, the angular resolution can surpass the limit value set by Abbe and Rayleigh. For the traditional slit-grooves or bull’s eye^20^, super-oscillation effect can be exploited to reduce the angular width. However, for OAM light, the side lobe at one direction may interfere with the main lobe at the opposite side, because of the fact that the main lobe of OAM is in a doughnut shape. Consequently, the side lobe should be confined to some extent so as to reduce the negative impacts on the main lobe. In the rest of this paper, we set the period of the grooves to be *p* = 470 nm, corresponding to a careful balance of the main lobe divergence angle and the side lobe level.

**Figure 3:**
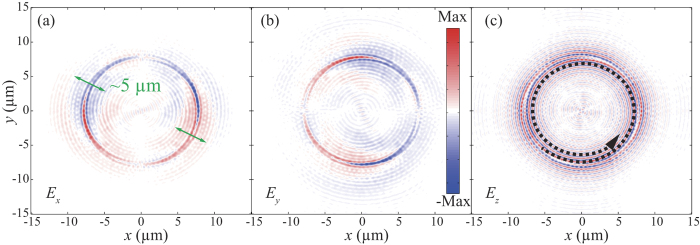
Spiral surface plasmon generated by the OAM light. (**a–c**) Real parts of the Electric fields polarized along the *x*, *y*, and *z* directions at the *xy* plane (*z* = 0 μm). The effective radiation aperture width is about 5 μm as indicated in (**a**). The spiral equal-phase line is indicated in (**c**).

Subsequently, we numerically calculated the electric fields distributions for the spiral surface plasmon for *l* = 2 using commercial software CST MWS. As shown in Fig. 3, the effective radiation aperture is about 5 μm, corresponding to a divergence angle of 5.7°. As depicted in Fig. 3(c), the SSPP is not propagating along a straight line any more. Instead, it propagates along a spiral line as determined by the topological charge of the incident OAM beam. Owing to the contribution of SSPP, the emitted OAM light becomes highly directional.

### Experimental validation

**Figure 4:**
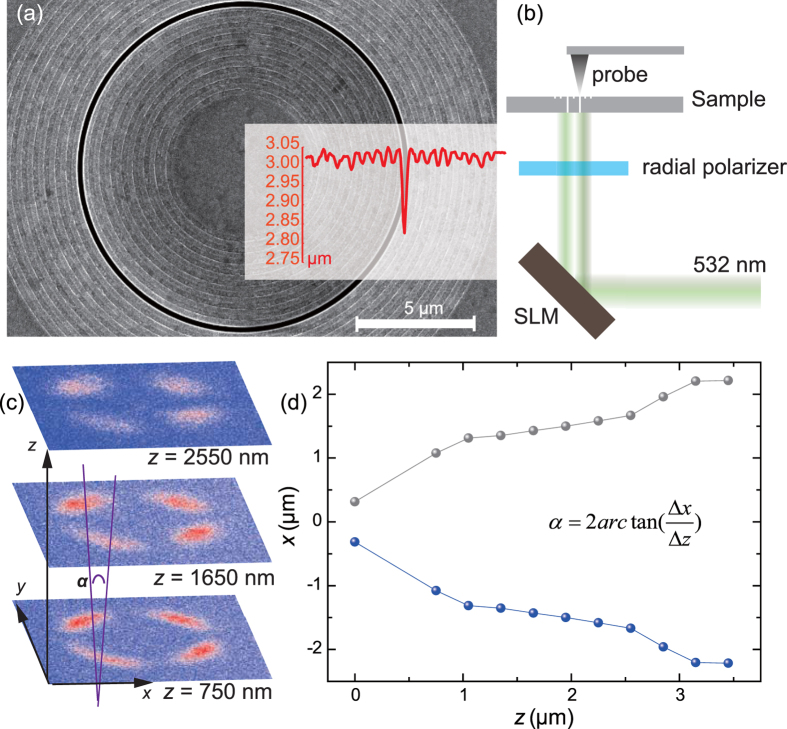
Experimental characterization of the collimated OAM. (**a**) Scanning electron microscope (SEM) image of the fabricated sample. Inset shows the atomic force microscope (AFM) image. (**b**) Setup of the measurement. (**c**) Measured near-field intensity at the *xy* plane for *l* = ± 2 at different distances away from the sample surface. The length and width along the *x* and *y* directions are both 20 μm. (**d**) Horizontal positions of the petals at different *z*.

In order to validate our proposal, a sample was fabricated by focused ion beam (FIB) milling on a 300 nm thick silver film deposited on a quartz (SiO_2_) substrate. The geometric parameters were chosen as the same as those in the theoretical simulations. Figure 4(a) shows the scanning electron microscope (SEM) image and atomic force microscope (AFM) image of the sample. The inset AFM image indicates that the groove depth is actually about 35 nm. The near field measurement was performed with a scanning near-field optical microscope (SNOM) (Fig. 4(b)). A linearly polarized laser beam at λ = 532 nm was first transformed into a Laguerre-Gaussian mode through a reflective SLM, and then converted to radial polarization with the help of a rotated waveplate formed by liquid crystal^36^. The finally collimated radially polarized OAM beam was then projected onto the backside of the sample, with the center of OAM beam superposed with that of the waveguide.

Although the OAM modes with different topological charges are intrinsic orthogonal to each other, all the OAM bemas share similar doughnut-shaped intensity patterns, except that the radius of the doughnut increases with increasing topological charge. In order to characterize the actual topological charge of the OAM beams, traditionally the interference pattern with plane or spherical wave should be exploited^8^,^12^. In fact, it is more convenient to use the petal-like interference pattern of two OAM beams with opposite topological charges^8^. The number of petals along the azimuthal direction is just the magnitude of the topological charge. In the experiment, we measured the near-field light intensity above the sample by using two superimposed waves carrying topological changes of *l* = ± 2. The measured results at *z *= 750 nm, 1650 nm, and 2550 nm are illustrated in Fig. 4(c), with small divergence as *z* increases. Figure 4(d) depicts the horizontal positions of the petals at different positions of *z*. The angular width was evaluated to be near α = 15.18° at *z* = 1μm, which is larger than the theoretical evaluated result. This difference may be stemmed from the variation of the dielectric constants of metal from the values in Ref. [34], since they are highly dependent on the evaporation environment, especially when the thickness is only hundreds of nanometers. Furthermore, the fabricated sample is still not perfect, as can be seen in the AFM image (the inset of Fig. 4(a)).

**Figure 5:**
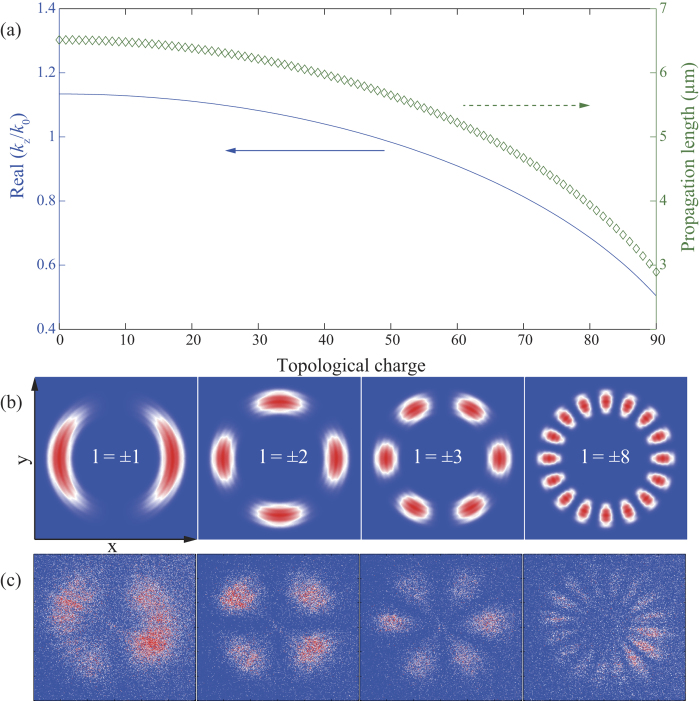
Dependence of the beaming effect on the topological charge. (**a**) Normalized propagation constants and propagation lengths for different topological charges. (**b**) Theoretically calculated and (**c**) experimentally measured petal beams at *z* = 10 μm. The corresponding topological charges are marked in (**b**). The length and width along the *x* and *y* directions in all panels are 20 μm.

One well-known property of the OAM waveguide is that there is a maximal topological charge for a given radius, above which the wave-vector along the propagating direction is imaginary, thus light is prohibited to propagate. For our coaxial OAM waveguide, the wavevector along the *z* direction follows^37^:
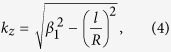
where *β*_1_ is the propagation constant of normal SPP inside a MIM waveguide. The characteristic equation of the fundamental TM mode is^23^:
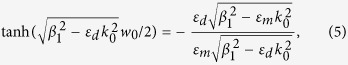
where *k*_0_ stands for the vacuum wavevector, *ε*_*m*_ and *ε*_*d*_ are the permittivities for the metal and dielectric media, and *w*_0_ is the width of the waveguide core. The propagation length, defined as the distance at which the intensity decrease to 1/2.71828 of its original value, can be calculated by 1/(2Im(*k*_z_)). As shown in Fig. 5(a), the propagation lengths in the designed waveguides are limited to be several microns for almost all topological charges.

When the guided OAM is coupled to the free-space, the dispersion relation at the output surface becomes:
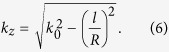
It seems that such a waveguide is able to support OAM with high topological charge approximating *l* = 90. Nevertheless, the practical topological charge may be much smaller than that obtained through equation (6). This is because that additional wavevector along the radial direction was ignored, which is stemming from the uncertainty principle Δ*k*_*r*_Δ*r* ≥ 2π, where Δ*r* is the effective width of the petal beam. Owing to the contribution of non-vanishing Δ*k*_*r*_, the actual propagation constant in free-space should be:
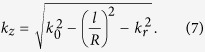


The above theoretical investigation only provides limited physical insight and guidance for the understanding of the OAM beaming effect. For a more accurate description, the beaming effect was calculated using vectorial diffraction theory^38^ by assuming an OAM beam with an effective aperture lies between *r*_1_ = 4.5 and *r*_2_ = 10.5. The results at *z *= 10 μm for *l* = ± 1, ± 2, ± 3 and ± 8 are plotted in Fig. 5(b), in good agreement with the experimental results shown in Fig. 5(c). The deterioration in the experimental performance for larger *l* may be resulted from the inaccuracy in the experimental process. For example, since there are no coupling grooves fabricated at the entrance size of the waveguide, only a small part of energy can be converted to the waveguide. Combining with the fact that limited energy can be collected by the probe of the SNOM, it is not strange that the signal-to-noise ratio is not high. In order to enhance the efficiency, coupling grooves at the entrance side can be exploited in future experiments.

## Discussion

In summary, we have demonstrated a plasmonic coaxial-waveguide-based scheme to transfer OAM beam with sub-wavelength mode confinement. Periodic and concentric grooves are used to coherently scatter the spiral surface plasmon to obtain collimated OAM beam. We found that the divergence angle reduction is accompanied with both the enlargement of the effective radiation aperture and the super-oscillatory superposition of diffracted electromagnetic fields. By employing the previous techniques used for surface plasmon engineering^20^,^39^, the angular distribution of OAM beam can be more precisely controlled by properly choosing the geometry of the grooves at the waveguide output surface. We also noted that there are various designs of micro- and nano-scale OAM generators reported in recent years^4^,^12^,^13^. By integrating these devices with the OAM waveguide, further enhancement of performance is foreseeable.

Although the plasmonic devices have a relatively large propagation loss, they are still feasible for on-chip applications^28^. For long-range propagation of OAM beam, cylindrical multilayers can be exploited to mimic the near-perfect reflection properties of metals and form all-dielectric coaxial waveguides^40^.

## Methods

### Numerical simulation

The commercial solvers CST microwave studio and COMSOL Multiphysics were used for the numerical evaluation of the 3D and 2D structure, respectively. Since the memory requirement for the 3D structure is much larger than its 2D counterpart, we only simulated a relatively small propagation distance (1 micron) along the *z*-direction. The electric fields at this output plane were then used as the input for a home-made solver based on vectorial plane wave expansion. The results of the 3D and 2D structures are comparable to each other.

### Sample fabrication

The sample was fabricated on a 1 mm thick quartz substrate. A 300-nm-thick Ag film was deposited on the cleaned substrate by magnetron sputtering in a sputter chamber. The sputter-deposition rate for Ag was *R*_Ag_ ≈ 0.83 nms^−1^. The slit and grooves were then milled on the Ag film using a Ga^+^ focused ion beam (FIB, FEI Helios Nanolab 650), and the accelerating voltage and current of the Ga^+^ beam were set as 30 KV and 24 pA, respectively.

### Measurement

The near field measurement was performed with a scanning near-field optical microscope (SNOM). A continuous-wave (CW) laser with vacuum wavelength of 532 nm (Cobolt Samba^TM^) was used as the light source. After passing through the beam extender and polarizing filter, the laser beam was exposed to a spatial light modulator (SLM). There are 1980 × 1080 pixels on SLM (Holoeye, Pluto, phase only SLM). The size of each pixel is 8 μm × 8 μm. We could independently control the phase of every pixel with a computer by creating a grey-scale map. Different grey levels (0–255) correspond to the different phases (0–2π). In this way, orbital angular momenta with different topological charges were added to the laser beam. Subsequently, a radial polarization converter (ARCoptix Switzerland) was used to convert the linear polarized OAM beam to radially polarized OAM. The propagation behavior of the OAM beam after passing through the designed sample were experimentally measured using a SNOM (NT-MDT, NTEGRA Solaris). When the probe (MF112_NTF) approached the surface of the sample, the elongation distance of the tube was recorded. After we turned off the feedback of the head, the tube would retract the same distance. So in this way, we could control the distance between the probe and the sample surface. The scanning size was chosen to be 20 μm × 20 μm, with points of 300 × 300, and the scanning velocity was 10 μm/s.

## Additional Information

**How to cite this article**: Pu, M. *et al.* Near-field collimation of light carrying orbital angular momentum with bull's-eye-assisted plasmonic coaxial waveguides. *Sci. Rep.* **5**, 12108; doi: 10.1038/srep12108 (2015).

## References

[b1] Fakonas J. S., Lee H., Kelaita Y. A. & Atwater H. A. Two-plasmon quantum interference. Nature Photon. 8, 317–320 (2014).

[b2] Pu M. *et al.* Spatially and spectrally engineered spin-orbit interaction for achromatic virtual shaping. Scientific Reports 5, 9822 (2015). 25959663 10.1038/srep09822 PMC4426594

[b3] Grier D. G. A revolution in optical manipulation. Nature 424, 810–816 (2003). 12917694 10.1038/nature01935

[b4] Ma X. *et al.* A planar chiral meta-surface for optical vortex generation and focusing. Scientific Reports 5, 10365 (2015). 25988213 10.1038/srep10365 PMC4437373

[b5] Allen L., Beijersbergen M. W., Spreeuw R. J. C. & Woerdman J. P. Orbital angular-momentum of light and the transformation of Laguerre-Gaussian laser modes. Phys. Rev. A 45, 8185–8189 (1992). 9906912 10.1103/physreva.45.8185

[b6] Novotny L., Bian R. X. & Xie X. S. Theory of nanometric optical tweezers. Phys. Rev. Lett. 79, 645–648 (1997).

[b7] Dholakia K., Reece P. & Gu M. Optical micromanipulation. Chem. Soc. Rev. 37, 42–55 (2008). 18197332 10.1039/b512471a

[b8] Franke-Arnold S., Allen L. & Padgett M. Advances in optical angular momentum. Laser Photon. Rev. 2, 299–313 (2008).

[b9] Tamburini F., Anzolin G., Umbriaco G., Bianchini A. & Barbieri C. Overcoming the Rayleigh criterion limit with optical vortices. Phys. Rev. Lett. 97, 163903 (2006). 17155396 10.1103/PhysRevLett.97.163903

[b10] Gibson G. *et al.* Free-space information transfer using light beams carrying orbital angular momentum. Opt. Express 12, 5448–5456 (2004). 19484105 10.1364/opex.12.005448

[b11] Edfors O. & Johansson A. J. Is orbital angular momentum (OAM) based radio communication an unexploited area? IEEE Trans. Antennas Propagat. 60, 1126–1131 (2012).

[b12] Yu N. *et al.* Light propagation with phase discontinuities: generalized laws of reflection and refraction. Science 334, 333–337 (2011). 21885733 10.1126/science.1210713

[b13] Cai X. *et al.* Integrated compact optical vortex beam emitters. Science 338, 363–366 (2012). 23087243 10.1126/science.1226528

[b14] Zhang S. *et al.* Chiral surface plasmon polaritons on metallic nanowires. Phys. Rev. Lett. 107, 096801 (2011). 21929259 10.1103/PhysRevLett.107.096801

[b15] Heeres R. W. & Zwiller V. Subwavelength focusing of light with orbital angular momentum. Nano letters 14, 4598–4601 (2014). 25051525 10.1021/nl501647t

[b16] Sun J. *et al.* Spinning light on the nanoscale. Nano Lett. 14, 2726–2729 (2014). 24697576 10.1021/nl500658n

[b17] Ma X. *et al.* Optical phased array radiating optical vortex with manipulated topological charges. Optics Express 23, 4873–4879 (2015). 25836522 10.1364/OE.23.004873

[b18] Wang Y. *et al.* Transfer of orbital angular momentum through sub-wavelength waveguides. Optics Express 23, 2857–2862 (2015). 25836146 10.1364/OE.23.002857

[b19] Chen C.-F. *et al.* Creating optical near-field orbital angular momentum in a gold metasurface. Nano letters 15, 2746–2750 (2015). 25798810 10.1021/acs.nanolett.5b00601

[b20] Lezec H. J. *et al.* Beaming light from a subwavelength aperture. Science 297, 820–822 (2002). 12077423 10.1126/science.1071895

[b21] Maier S. A. *et al.* Local detection of electromagnetic energy transport below the diffraction limit in metal nanoparticle plasmon waveguides. Nature Mater. 2, 229–232 (2003). 12690394 10.1038/nmat852

[b22] Luo X. & Ishihara T. Surface plasmon resonant interference nanolithography technique. Appl. Phys. Lett. 84, 4780–4782 (2004).

[b23] Pu M. *et al.* Directional coupler and nonlinear Mach-Zehnder interferometer based on metal-insulator-metal plasmonic waveguide. Opt. Express 18, 21030–21037 (2010). 20940998 10.1364/OE.18.021030

[b24] Yi J.-M., Cuche A., Devaux E., Genet C. & Ebbesen T. W. Beaming visible light with a plasmonic aperture antenna. ACS Photon. 1, 365–370 (2014). 10.1021/ph400146n PMC4270416 25540811

[b25] Luo X. Principles of electromagnetic waves in metasurfaces. Science China-Physics, Mechanics & Astronomy 58, 094201 (2015).

[b26] Gorodetski Y., Drezet A., Genet C. & Ebbesen T. W. Generating far-field orbital angular momenta from near-field optical chirality. Phys. Rev. Lett. 110, 203906 (2013). 25167414 10.1103/PhysRevLett.110.203906

[b27] Rüting F., Fernández-Domínguez A. I., Martín-Moreno L. & García-Vidal F. J. Subwavelength chiral surface plasmons that carry tuneable orbital angular momentum. Phys. Rev. B 86, 075437 (2012).

[b28] De Waele R., Burgos S. P., Polman A. & Atwater H. A. Plasmon dispersion in coaxial waveguides from single-cavity optical transmission measurements. Nano Lett. 9, 2832–2837 (2009). 19603794 10.1021/nl900597z

[b29] Peng Y., Wang X. & Kempa K. TEM-like optical mode of a coaxial nanowaveguide. Opt. Express 16, 1758–1763 (2008). 18542255 10.1364/oe.16.001758

[b30] Lerman G. M., Yanai A. & Levy U. Demonstration of nanofocusing by the use of plasmonic lens illuminated with radially polarized light. Nano letters 9, 2139–2143 (2009). 19391611 10.1021/nl900694r

[b31] Si G. *et al.* Annular aperture array based color filter. Applied Physics Letters 99, 033105 (2011).

[b32] Liu Y. J. *et al.* Light‐driven plasmonic color filters by overlaying photoresponsive liquid crystals on gold annular aperture arrays. Advanced Materials 24, OP131–OP135 (2012). 22438069 10.1002/adma.201104440

[b33] Bozinovic N. *et al.* Terabit-scale orbital angular momentum mode division multiplexing in fibers. Science 340, 1545–1548 (2013). 23812709 10.1126/science.1237861

[b34] Palik E. D. Handbook of Optical Constants of Solids. (Academic press, 1985).

[b35] Di Francia G. T. Super-gain antennas and optical resolving power. G. Suppl. Nuovo Cim. 9, 426–438 (1952).

[b36] Stalder M. & Schadt M. Linearly polarized light with axial symmetry generated by liquid-crystal polarization converters. Opt. Lett. 21, 1948–1950 (1996). 19881855 10.1364/ol.21.001948

[b37] Schmidt M. A., Sempere L. N. P., Tyagi H. K., Poulton C. G. & Russell P. S. J. Waveguiding and plasmon resonances in two-dimensional photonic lattices of gold and silver nanowires. Phys. Rev. B 77, 033417 (2008).

[b38] Liu T., Tan J., Liu J. & Wang H. Vectorial design of super-oscillatory lens. Opt. Express 21, 15090–15101 (2013). 23842296 10.1364/OE.21.015090

[b39] Wang C., Du C. & Luo X. Refining the model of light diffraction from a subwavelength slit surrounded by grooves on a metallic film. Phys. Rev. B 74, 245403 (2006).

[b40] Ibanescu M., Fink Y., Fan S., Thomas E. L. & Joannopoulos J. D. An all-dielectric coaxial waveguide. Science 289, 415–419 (2000). 10903194 10.1126/science.289.5478.415

